# Signatures of cytoplasmic proteins in the exoproteome distinguish community- and hospital-associated methicillin-resistant *Staphylococcus aureus* USA300 lineages

**DOI:** 10.1080/21505594.2017.1325064

**Published:** 2017-05-05

**Authors:** Solomon A. Mekonnen, Laura M. Palma Medina, Corinna Glasner, Eleni Tsompanidou, Anne de Jong, Stefano Grasso, Marc Schaffer, Ulrike Mäder, Anders R. Larsen, Heidi Gumpert, Henrik Westh, Uwe Völker, Andreas Otto, Dörte Becher, Jan Maarten van Dijl

**Affiliations:** a Department of Medical Microbiology, University of Groningen, University Medical Center, Groningen, Groningen, The Netherlands; b Interfaculty Institute for Genetics and Functional Genomics, University Medicine Greifswald, Greifswald, Germany; c Department of Molecular Genetics, University of Groningen, Groningen Biomolecular Sciences and Biotechnology Institute, Groningen, The Netherlands; d National Center for Antimicrobials and Infection Control, Statens Serum Institut, Copenhagen, Denmark; e Department of Clinical Microbiology, Hvidovre University Hospital, Hvidovre, Denmark; f Department of Clinical Medicine, Faculty of Health, University of Copenhagen, Copenhagen, Denmark; g Institut für Mikrobiologie, Ernst-Moritz-Arndt-Universität Greifswald, Greifswald, Germany

**Keywords:** community, epithelial cells, exoproteome, hospital, moonlighting, MRSA, protein secretion, *Staphylococcus*, USA300, virulence factor

## Abstract

Methicillin-resistant *Staphylococcus aureus* (MRSA) is the common name for a heterogeneous group of highly drug-resistant staphylococci. Two major MRSA classes are distinguished based on epidemiology, namely community-associated (CA) and hospital-associated (HA) MRSA. Notably, the distinction of CA- and HA-MRSA based on molecular traits remains difficult due to the high genomic plasticity of *S. aureus*. Here we sought to pinpoint global distinguishing features of CA- and HA-MRSA through a comparative genome and proteome analysis of the notorious MRSA lineage USA300. We show for the first time that CA- and HA-MRSA isolates can be distinguished by 2 distinct extracellular protein abundance clusters that are predictive not only for epidemiologic behavior, but also for their growth and survival within epithelial cells. This ‘exoproteome profiling’ also groups more distantly related HA-MRSA isolates into the HA exoproteome cluster. Comparative genome analysis suggests that these distinctive features of CA- and HA-MRSA isolates relate predominantly to the accessory genome. Intriguingly, the identified exoproteome clusters differ in the relative abundance of typical cytoplasmic proteins, suggesting that signatures of cytoplasmic proteins in the exoproteome represent a new distinguishing feature of CA- and HA-MRSA. Our comparative genome and proteome analysis focuses attention on potentially distinctive roles of ‘liberated’ cytoplasmic proteins in the epidemiology and intracellular survival of CA- and HA-MRSA isolates. Such extracellular cytoplasmic proteins were recently invoked in staphylococcal virulence, but their implication in the epidemiology of MRSA is unprecedented.

## Introduction

*Staphylococcus aureus* is a wide-spread commensal bacterium, but also a notoriously drug-resistant pathogen that causes a wide range of diseases, varying from mild skin infections to life-threatening invasive diseases.^1^ About 20–30% of the healthy human population is known to carry *S. aureus*, the anterior nares being the preferred niche.^2^

Since the clinical implementation of antibiotics, *S. aureus* has acquired a range of resistance traits through mutations and horizontal gene transfer. This has culminated in the emergence of methicillin-resistant *S. aureus* (MRSA), a major healthcare problem world-wide.^3,4^ The emergence of MRSA is a particularly worrisome development since it is associated with increased morbidity and mortality, especially if very young, immune-compromised or elderly individuals are infected.^5,6^ Moreover, no effective vaccine against MRSA is currently available.^7-9^

Two major classes of MRSA are currently distinguished based on their epidemiology, namely community-associated (CA) and hospital-associated (HA) MRSA. CA-MRSA is mainly a threat to healthy individuals, causing in particular skin and soft tissues infections, but also serious invasive infections such as pneumonia and osteomyelitis.^10-13^ In contrast, HA-MRSA infections are associated with prolonged hospitalization, stay in intensive care units, hemodialysis, surgery, and long-term exposure to antibiotics.^14^

Molecular markers for high-confidence distinction between CA- and HA-MRSA isolates are urgently needed in the prevention and control of hospital outbreaks. Different DNA typing methods, such as pulsed-field gel electrophoresis (PFGE) and *Staphylococcus* protein A (*spa*) typing have been used to differentiate between these 2 classes of MRSA.^15^ This was so far feasible, because particular *S. aureus* lineages with distinct sequence types are associated with the CA- or HA-associated behavior. In addition, particular virulence genes (e.g. for the Panton-Valentin leukocidin; PVL), the arginine catabolic mobile element (ACME), and mobile genetic elements carrying the *mecA* gene for methicillin resistance are used to distinguish CA- and HA-MRSA.^11-14,16,17^ However, such DNA-based typing methods do not allow easy distinction between closely related CA- and HA-MRSA lineages, because the causative molecular features have remained largely enigmatic. For instance, PFGE assigns CA-MRSA isolates with the *spa* type t008 and HA-MRSA isolates with the *spa* type t024 to the same USA300 lineage.^18^ Likewise, *spa* typing has insufficient discriminatory power to distinguish closely related CA and HA isolates as it assigns CA-USA300 isolates with the multi-locus sequence type ST8 and more distantly related HA isolates with the sequence type ST8 to the same *spa* type t008.^18^ Nevertheless, we have previously shown that a multiple-locus variable number tandem repeat fingerprinting (MLVF) approach may distinguish these highly related *S. aureus* isolates.^19^

An important challenge for the clinic is that *S. aureus* types previously regarded as CA, such as USA300 and the European ST80 clone, are becoming common hospital pathogens causing outbreaks.^18,20-22^ Clearly, an increasing prevalence of CA-MRSA in the community makes it harder to exclude the respective lineages from hospitals, because they can be carried into the hospitals by MRSA-positive patients, healthcare workers and visitors. Furthermore, it is conceivable that these bacteria have acquired, either before or after entry into the hospital environment, properties that facilitate their spread in this setting. The latter view would be supported by the observation that the closely related USA300 isolates with *spa* types t008 and t024 display different epidemiology.^18^

The distinction of CA- and HA-MRSA at the molecular level is challenging, because many factors may contribute to bacterial epidemiological behavior, not in the last place interactions with the human host. High-throughput analytical ‘omics’ approaches, especially genomics and proteomics, are particularly suitable for exploring such multi-factorial behavior since they allow the definition of feature- or condition-specific signatures.^23,24^ Furthermore, proteomics applied to bacterial pathogens grown under infection-mimicking conditions is a powerful tool for investigating different lineage- or type-specific patterns of gene expression.^25^ In the context of infection-related research, it is important to focus special attention on the extracellular proteome (‘exoproteome’) as it represents the main reservoir of virulence factors that are first in interacting with the human host.^26,27^ Specifically, secreted toxins and other virulence factors of *S. aureus* contribute to tissue damage, host invasion, and evasion of the host's immune responses.^28,29^ Thus, proteomics has a high potential for identifying diagnostic biomarkers, and novel vaccine or drug targets.^30^

To obtain a better understanding of the molecular differences between CA- and HA-MRSA, the present study was aimed at a global comparative genome and exoproteome analysis of 12 MRSA isolates belonging to the USA300 lineage as defined by PFGE. As these isolates were all collected from Denmark (DK), we refer to them as the CA^DK^ and HA^DK^ isolates. Specifically, the CA^DK^ group had the sequence type ST8, the *spa* type t008 and was PVL-positive, whereas the HA^DK^ group was characterized by the sequence type ST8 and the *spa* type t024.^18,21^ As a control group, we also investigated the exoproteomes of 3 HA-MRSA isolates from the Dutch (NL) - German (DE) border region, here referred to as HA^NL-DE^, which have the sequence type ST8, and *spa* type t008 or t024.^19^ The genomes of all 15 isolates were sequenced, and their extracellular proteins were analyzed by liquid chromatography and mass spectrometry (LC-MS). In brief, CA and HA isolates could be distinguished to some extent by the accessory genome. More importantly, a principal component analysis (PCA) of the exoproteome MS data clustered the 15 investigated isolates into 2 groups that match their different epidemiological behavior.

## Results

### Comparative genomic analysis

**Figure 1.**
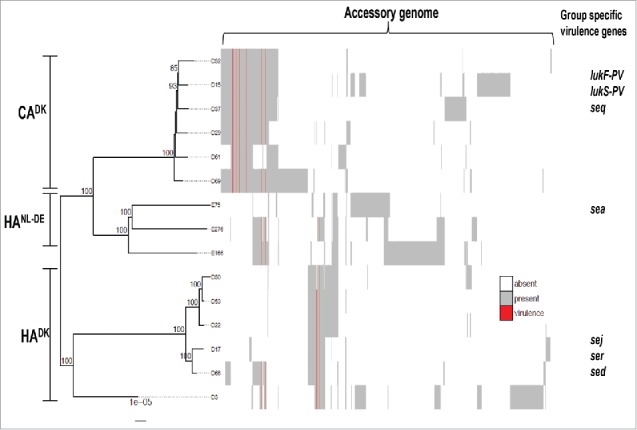
Phylogenetic tree and accessory genomes of all 15 investigated CA^DK^, HA^DK^ and HA^NL-DE^ isolates. The tree is midpoint rooted and bootstrap support >70% is indicated on the branches. The heatmap to the right of the phylogenetic tree illustrates the accessory genome. The columns of the heatmap are hierarchically clustered based on the presence/absence of genes. Known virulence genes are indicated in red. Examples of virulence genes that are exclusively present in one of the 3 groups are indicated as group-specific virulence genes.

Whole genome sequence analysis was performed to determine the genomic similarities and differences of all 15 investigated isolates. A phylogenetic tree based on the core genome of the isolates showed that the 6 CA^DK^, and 5 of the 6 HA^DK^ isolates formed 2 distinct clusters (Fig. 1). One HA^DK^ isolate (D3) showed a more distant relationship with the other HA^DK^ isolates. Furthermore, the 3 HA^NL-DE^ isolates formed a separate cluster that is closer to the CA^DK^ than the HA^DK^ isolates. In addition to the phylogenetic analysis, a comparative analysis of the accessory genomes of the isolates was performed, which is presented as a heatmap in Fig. 1. As illustrated in the heat map, the CA isolates have overall more accessory genes than the HA isolates. Perhaps more importantly, the clustering of accessory genes is indicative of a separation between the CA and HA isolates, irrespective of the geographical origin of the HA isolates. This separation is also reflected in the presence or absence of several known virulence genes (red lines in Fig. 1), such as the PVL-encoding genes *lukF* and *lukS* that were exclusively found in the CA isolates, and the enterotoxin-encoding genes *sea, sed, sej*, and *ser* that were only present in the investigated HA isolates (Table S1)*.* Of note, PVL is often used as a marker for CA-MRSA and enterotoxin genes appear to be rare in CA isolates of the USA300 lineage,^31^ but a possible association of enterotoxin genes with HA behavior would be novel.

Both CA- and HA-MRSA isolates carried a *norA* gene that provides resistance to fluoroquinolones, and *mecA* and *blaZ* genes for β-lactam resistance (Table S2). Genes potentially providing resistance to macrolides, lincosamides and streptogramin B (*msr*(*A*)), aminoglycosides (*aph*(3′)-III), and macrolides (*mph*(*C*)) were exclusively identified in the CA-MRSA isolates, whereas *erm*(*A*) and *spc* that provide resistance to macrolides and aminoglycosides, respectively, were exclusively identified among the HA-MRSA isolates (Table S2). Altogether, the CA-MRSA isolates carried more (potential) antimicrobial resistance genes than the investigated HA-MRSA isolates.

### Unique and shared exoproteins

**Figure 2.**
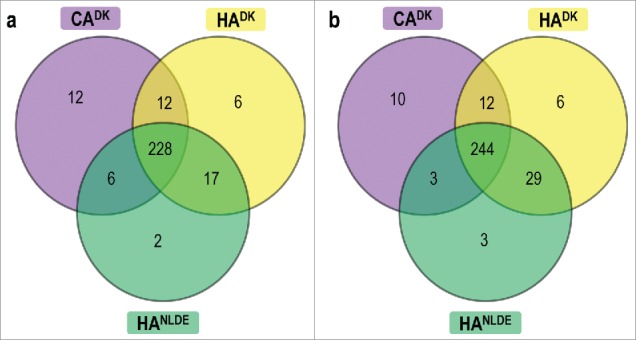
Shared and uniquely identified proteins in CA^DK^, HA^DK^ and HA^NL-DE^ *S. aureus* isolates. The Venn diagrams relate to cells in the exponential (a) and stationary (b) growth phases. The numbers of commonly and uniquely identified proteins of the different groups of isolates are indicated.

To characterize the exoproteomes of the 15 MRSA isolates, they were cultured in RPMI medium since a recent study showed that global gene expression profiles of *S. aureus* cells grown in RPMI or human plasma are highly similar.^24^ Samples were withdrawn for exoproteome analyses at mid-exponential growth phase and 90 min after entry into the stationary phase. No major differences in the growth curves of the 15 MRSA were observed (data not shown). As shown by gel-free mass spectrometry, a total number of 409 unique proteins was identified from the 15 exoproteome samples of exponentially grown isolates. Similarly, a total number of 458 unique proteins was identified from the 15 exoproteome samples generated from stationary phase cultures. Proteins were considered for further analyses when they were present in at least 50% of the isolates of a particular group, i.e. when a protein was present in 3 out of the 6 isolates in CA^DK^ and HA^DK^, and in 2 out of 3 isolates in HA^NL-DE^. Thus, 283 and 307 unique proteins identified in the exponential or stationary phase samples, respectively, were included in the subsequent analyses (Table S3). The majority of these proteins was shared by all 3 groups both in the exponential (Fig. 2a) and stationary (Fig. 2b) growth phases. Importantly, there are more proteins shared by the HA^DK^ and HA^NL-DE^ isolates than by the HA^DK^ or HA^NL-DE^ isolates and CA^DK^ isolates. This implies that, in terms of exoprotein production, the 2 groups of HA isolates are more closely related with each other than the HA and CA isolates. Furthermore, unique proteins ranging from 2 to 12 proteins in the exponential growth phase, and 3 to 10 proteins from the stationary growth phase, which were specific to only one of the 3 groups of isolates were identified (Fig. 2a, b). Together, these data show that the majority of extracellular proteins of the CA^DK^, HA^DK^ and HA^NL-DE^ is common. Yet, a subset of the exoproteins appears to be specific for each of the 3 groups of isolates.

### Predicted sub-cellular localization of identified exoproteins

**Figure 3.**
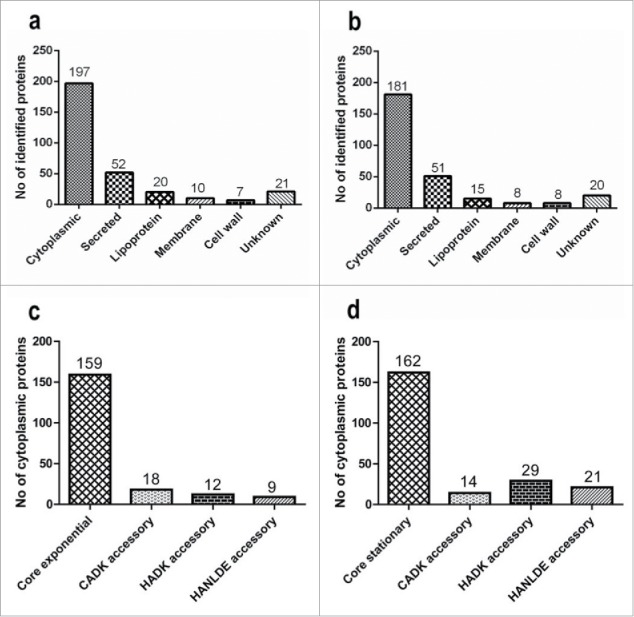
Predicted subcellular localization of identified extracellular proteins. The predicted subcellular localization of all 494 identified extracellular proteins is shown for cells in the exponential (a) and stationary (b) growth phases. Panels (c) and (d), respectively, highlight the appearance of predicted cytoplasmic core and accessory cytoplasmic proteins in the growth medium of the CA^DK^, HA^DK^ and HA^NL-DE^ isolates in the exponential and stationary growth phases. The numbers of proteins identified in each category are indicated at the top of the bars.

Bacterial exoproteomes are known to contain proteins that are actively secreted and proteins that are liberated from the cells through (auto-)lysis or other unidentified ‘non-classical secretion’ mechanisms.^27,32-37^ These proteins can be distinguished through signal peptide predictions, which is relevant as most known virulence factors contain signal peptides to direct their export from the cytoplasm.^26^ Thus, we predicted the sub-cellular localization of proteins that were identified by MS. The vast majority of the proteins identified in the exoproteomes of the isolates in the exponential and stationary growth phases were assigned to the class of cytoplasmic proteins followed by secreted proteins, lipoproteins, cytoplasmic membrane proteins and cell wall-associated proteins (Fig. 3a, b). Notably, in the exponential growth phase, the numbers of accessory exoproteins that were predicted as cytoplasmic were higher in the CA^DK^ group than in the HA^DK^ and HA^NL-DE^ groups (Fig. 3c). Conversely, in the stationary phase, the numbers of accessory exoproteins predicted as cytoplasmic were higher among the HA^DK^ and HA^NL-DE^ groups than in the CA^DK^ group (Fig. 3d). For exoproteins with a predicted localization in the membrane (i.e., membrane- and lipoproteins) or cell wall no major differences were observed in the 3 groups, irrespective of the growth phase (data not shown). Lastly, higher numbers of predicted secretory proteins were identified in growth media of the HA group than the CA group in both growth phases. Altogether, these data imply that the investigated CA and HA isolates are similar in terms of the predicted localization of their exoproteins. Nonetheless, the main distinction among these groups was the time point at which cytoplasmic proteins are liberated from the cells.

### Relative extracellular abundance of known and putative virulence factors

**Figure 4.**
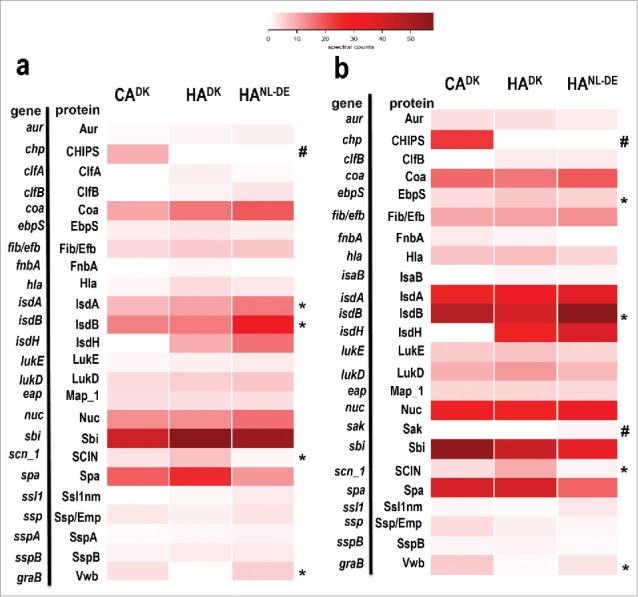
Heat map analysis of quantified extracellular virulence factors. The normalized spectral counts of known extracellular virulence factors identified by Mass Spectrometry in growth media of the 3 groups of isolates are graphically represented as colored heat maps. Each heat map includes 3 columns representing each of the 3 groups of the isolates. Of note, each column of CA^DK^ and HA^DK^ isolates is based on the average of 6 different isolates each analyzed in duplicate, and the HA^NL-DE^ column is based on the average of 3 different isolates each analyzed in duplicate. Each row represents a particular protein. Panels (a) and (b) represent known virulence factors of *S. aureus* as identified in the growth medium fractions of cells in the exponential and stationary growth phases, respectively. *Statistically significant differences in relative abundance of the proteins marked between the groups; ^#^ Proteins present in one group of isolates only.

To obtain more comprehensive insights in the possible differences in the levels of known extracellular virulence factors, we assessed their relative abundance for the different investigated isolates*.* Detailed evaluation of the normalized spectral counts showed differential and similar expression levels for 24 virulence factors among the 3 groups of isolates both in the exponential growth phase (Fig. 4a), and in the stationary growth phase (Fig. 4b). Of note, neither PVL nor enterotoxins that were identified as potentially distinguishing features for CA and HA isolates based on the genome sequence were detectable in the extracellular proteome. On the other hand, statistically significantly different levels of the IsdA, IsdB, SCIN and Vwb proteins were identified in the growth media of exponentially growing isolates, and the same was true for the Ebps, IsdB, SCIN and Vwb proteins in the growth media of stationary growing isolates (Fig. 4a, b, Table S4).

**Figure 5.**
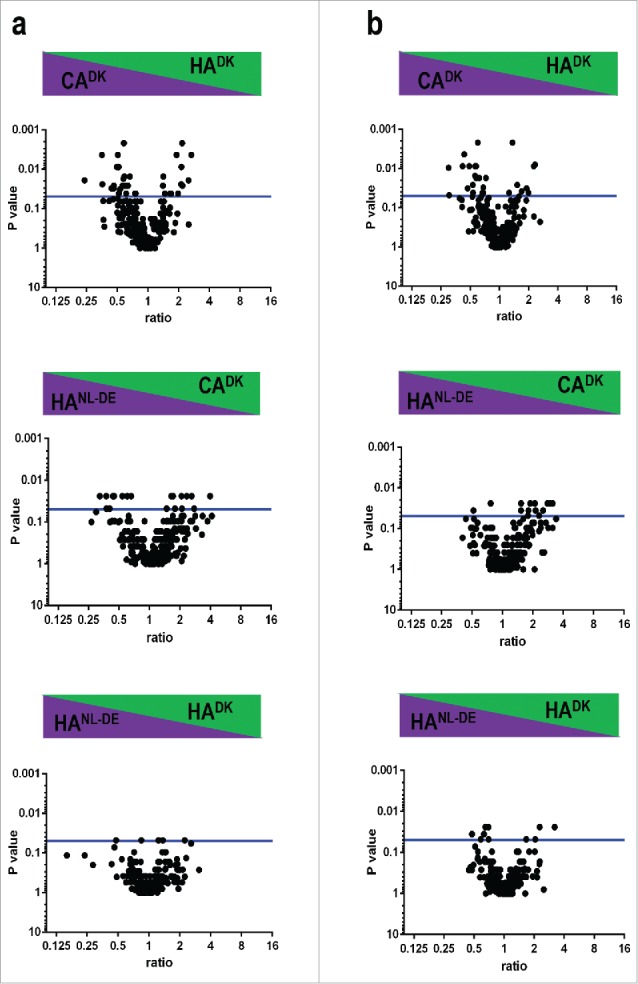
Differences in relative extracellular protein abundance in CA^DK^, HA^DK^ and HA^NL-DE^ isolates. Statistically significant differences in the relative abundance of identified extracellular proteins are presented in volcano plots for samples collected in the exponential (a) and stationary (b) growth phases. Horizontal blue lines indicate a p-value threshold of 0.05.

The relative amounts of individual secreted exoproteins are likely important for the behavior of the respective *S. aureus* isolate, especially where this concerns secreted toxins or immune evasion factors. Therefore, we determined the relative abundance of proteins in the 3 groups of isolates from the normalized spectral counts of proteins. A volcano plot was used to present the proteins that were detectable at statistically significantly higher or lower levels among the 3 groups of isolates during both the exponential and stationary growth phases. From the total of 283 proteins identified in samples collected in the exponential growth phase, a relatively large number of proteins was present at statistically significantly different levels when the CA^DK^ and the 2 HA isolate groups (*i.e* HA^DK^ and HA^NL-DE^) were compared, and this difference was larger than the difference between the HA^DK^ and HA^NL-DE^ isolates (Fig. 5a; Table S2). A similar pattern was observed for the samples harvested during the stationary phase (Fig. 5b). Additionally, some proteins were exclusively present in one group of isolates, e.g., the chemotaxis inhibitory protein (CHIPS) was identified only in CA^DK^, the enterotoxin type D only in HA^DK^, and the enterotoxin type A only in HA^NLDE^ isolates, and this applied both to exponential and stationary phase growth medium samples (Table S5). Together, these data show differences in the relative abundance of extracellular proteins at statistically significant levels in all the 3 groups of isolates, but especially for the CA and HA isolates.

### Levels of mRNA for selected exoproteins

**Figure 6.**
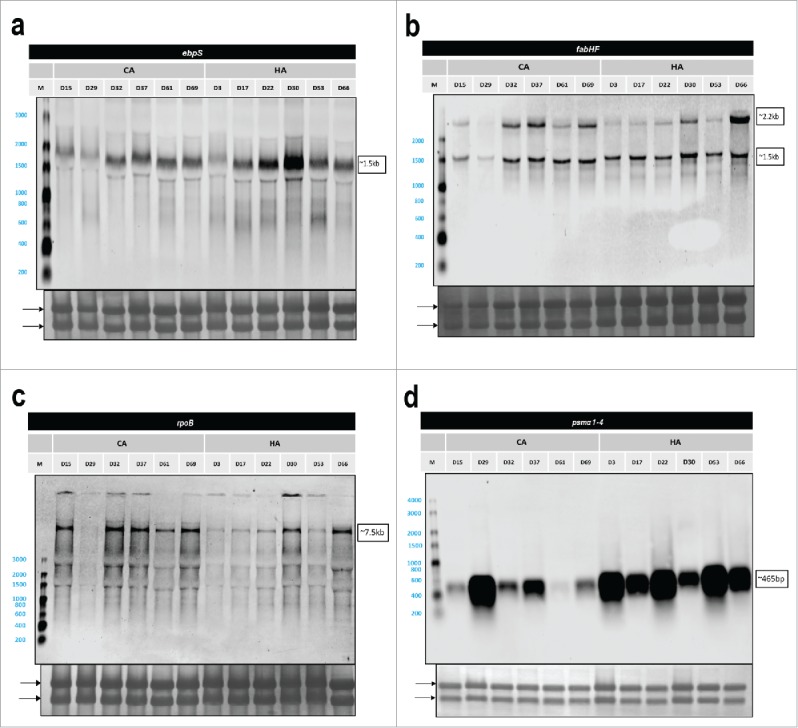
Northern blotting analysis of selected genes. Arrows mark the positions of 23S- and 16S-rRNA bands on the methylene blue stained membranes. Sizes of specific transcripts are indicated on the right side of each display. In case of *fabHF* 2 bands were detected, the larger band of ∼2.2 kb representing a *fabHF* transcript and the lower band of ∼1.5 kb only *fabF*. (a) *ebps*, (b) *fabHF*, (c) *rpoB*, and (d) *psmα1*–4.

The abundance of a bacterial exoprotein reflects the net result of transcription of the respective gene, mRNA translation, translocation of the precursor protein across the membrane, post-translocational folding of the protein into a stable conformation, cell wall passage and the protein's stability in the bacterial extracellular milieu. This implies that extracellular protein abundance is not always linearly correlated with the transcript levels. Yet, mRNA levels are major determinants for protein expression levels. Therefore, a Northern blotting analysis was performed to assess whether there is a possible correlation between transcript levels and extracellular protein abundance. Specifically, we compared the transcript levels for a secreted virulence factor (*ebpS*) and 2 cytosolic proteins (*fabF* and *rpoB*). Consistent with the MS data, in the stationary phase, the mRNA level of *ebpS* was higher in HA isolates than in the CA isolates (Fig. 6a), whereas the mRNA levels of *fabF* and *rpoB* were higher in the CA isolates compared with the HA isolates (Fig. 6b, c). Of note, the *fabF* and *rpoB* mRNA levels in the CA^DK^ isolate D29 were more similar to the respective mRNA levels in HA^DK^ isolates than to those in the CA^DK^ isolates. On the other hand, 2 of HA^DK^ isolates (D30 and D66), displayed *fabF* and *rpoB* mRNA levels comparable to those observed for the CA^DK^ isolates.

Northern blotting analyses can also provide information on the expression of genes for which the encoded proteins were not covered by the proteome analysis. Since phenol-soluble modulins (PSMs) are particularly relevant for virulence, but notoriously difficult to identify by proteomics due to their small size, we investigated the *psmα1–4* mRNA levels by Northern blotting. In the stationary growth phase, the *psmα1–4* mRNA levels were higher in most of the HA^DK^ isolates than in the CA^DK^ isolates (Fig. 6d). However, also the CA^DK^ isolate D29 showed a relatively high level of *psmα1*–4 mRNA that was comparable to the *psmα1*–4 mRNA levels in the HA^DK^ isolates (Fig. 6d). Based on these Northern blotting data, the proteomics data were reassessed with less stringent criteria where we considered also proteins identified with only one peptide. Thus, we were able to identify both the PSMβ1 and PSMβ2 proteins in medium fractions of 5 out of the 6 HA^DK^ isolates grown to stationary phase. Of note, the same was true for the D29 CA^DK^ isolate. These findings are fully consistent with the relative mRNA levels detected by Northern blotting.

### Clustering of CA and HA isolates based on exoproteome abundance signatures

**Figure 7.**
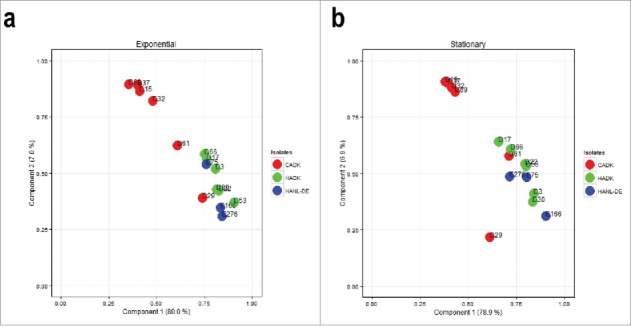
Principal component analysis (PCA) of the normalized spectral counts of identified extracellular proteins. Two-dimensional PCA plots are displayed for growth medium samples from the 15 CA^DK^, HA^DK^ and HA^NL-DE^ isolates in the (a) exponential growth phase and (b) stationary growth phase.

Principal component analysis (PCA) was performed to assess the overall relationships between the different investigated isolates in terms of their exoproteome profiles. Of note, this PCA was based on the normalized spectral counts of proteins that were produced by all 3 groups of isolates, specifically 283 proteins from exponentially growing bacteria, and 308 proteins from bacteria in the stationary growth phase. Importantly, the PCA analysis revealed that the CA- and HA-MRSA isolates clustered in 2 distinct groups based on the ‘exoprotein abundance signatures’ where the HA cluster included both the HA^NL-DE^ and the HA^DK^ isolates (Fig. 7a, b) irrespective of their geographical origin. Yet, the HA cluster included 2 CA^DK^ isolates (D29 and D61), whose exoprotein abundance signatures apparently resemble those of the analyzed HA isolates.

The PCA analysis in Fig. 7 was based on all identified extracellular proteins, including proteins with different predicted subcellular localizations. To assess whether the discriminating information relates to proteins with a particular predicted localization site, PCA analysis was performed on: i, predicted cytoplasmic proteins alone, ii, all identified proteins except the predicted cytoplasmic proteins, and iii, all identified proteins except the predicted cytoplasmic proteins and the proteins of unknown localization. Unexpectedly, the distinguishing information was primarily associated with the predicted cytoplasmic proteins Table S6).

Voronoi treemaps can be applied to link quantitative proteomic data and functional classifications. Thus, we used Voronoi treemaps to characterize the extracellular proteins identified for the 3 groups of isolates. The biologic functions of the identified proteins were mainly related to protein biosynthesis, carbohydrate and carbon metabolism, oxidative stress, and adhesion (Fig. S1a, S1b). Of note, adhesion-associated extracellular proteins were somewhat more pronounced among the HA- than the CA isolates in the exponential growth phase (Fig. S1a). Conversely, adhesion-related extracellular proteins were more prominently present in CA- than in HA isolates in the stationary growth phase (Fig. S1b). Despite the fact that there were unique proteins identified in each of the 3 groups of isolates, no major differences in the overall functions of the identified extracellular proteins were observed.

### Differences in staphylococcal survival within epithelial cells

**Figure 8.**
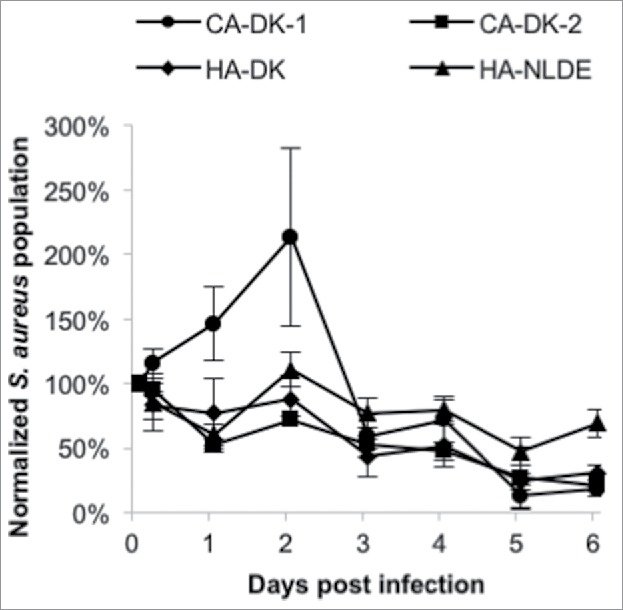
Survival of CA and HA isolates internalized by 16HBE14o- bronchial epithelial cells. Averaged survival curves are shown for the CA^DK^, HA^DK^ and HA^NL-DE^ isolates, where the CA^DK^ isolates are separated into 2 groups in accordance with their exoprotein abundance signatures in Fig. 8 (CA-DK-1 includes isolates D15, D32, D37 and D69; CA-DK-2 includes isolates D29 and D61).

Since the PCA analysis of exoproteins grouped the studied *S. aureus* isolates into 2 distinct clusters, we asked the question whether these groups might interact differently with human host cells. A bronchial epithelial cell line (16HBE14o-) was selected for this purpose, because CA-MRSA isolates have been implicated in severe respiratory infections among healthy individuals from the community. This fact might indicate a superior ability of this group of bacteria to interact with airway cells. Furthermore, the 16HBE14o- epithelial cell line forms a confluent layer that allows the monitoring of infecting bacteria over several days. Thus, *in vitro* cultured 16HBE14o- epithelial cells were infected with the CA^DK^, HA^DK^ and HA^NL-DE^ isolates, and the subsequent binding, internalization and intracellular survival of staphylococci were assessed through counting by flow cytometry. While the staphylococcal isolates did not show major differences in the internalization rate into the epithelial cells, there was a marked difference in post-internalization growth and survival. As shown in Fig. 8 and Fig. S2b, 4 CA^DK^ isolates (D15, D32, D37, D69) were able to multiply inside the epithelial cells during the first 2 d post infection, after which the population size decreased. In contrast, the HA^DK^ isolates did not multiply with the exception of isolate D17, which showed a slight increase, comparable to the CA^DK^ isolate D69 (Fig. 8). Of note, the CA^DK^ isolates D29 and D61, which were grouped with the HA^DK^ isolates in the exoproteome PCA analysis (Fig. 7), displayed similar intracellular behavior as the 5 HA^DK^ isolates D03, D22, D30, D53 and D66. Notably, all the 3 HA^NL-DE^ isolates showed a similar intracellular behavior as the HA^DK^ isolates. These findings imply that the distinction of the investigated HA and CA isolates based on differences in their exoproteomes, is reflected in their growth and survival behavior upon internalization of human bronchial epithelial cells.

### Discussion

The serious threat that MRSA represents for hospitalized patients demands an accurate and reliable method of distinction between CA- and HA-MRSA isolates for the purpose of prevention and control of outbreaks. In the present study, we explored the feasibility of applying a combined genome and proteomics-based approach to distinguish MRSA isolates with different epidemiological behavior. To facilitate the interpretation of the complex data, we used a set of genetically closely related CA- and HA-MRSA isolates with the sequence type ST8. Altogether, our findings revealed several distinguishing features between the investigated CA and HA isolates at the levels of the accessory genome and the exoproteome.

Since exoproteins have major roles in staphylococcal colonization of the host and virulence, we compared the exoproteome profiles of 3 genetically similar, but epidemiologically unrelated groups of MRSA isolates, i.e., the CA^DK^, HA^DK^ and HA^NL-DE^ isolates. Noticeably, most of identified exoproteins was shared by all the 3 groups of isolates. However, some of the identified exoproteins were unique for a particular group of isolates, while the abundance of several common proteins varied among the 3 groups of isolates. This probably reflects the fact that the *S. aureus* exoproteome is heterogeneous due to this organism's genomic plasticity.^26,27^ Nonetheless, the investigated HA^NL-DE^ isolates shared more similarities with respect to accessory virulence genes and actually detected exoproteins with the HA^DK^ isolates than with the CA^DK^ isolates, even though their core genome was more closely related to that of the CA^DK^ isolates. The latter finding is in line with previously reported observations that genetically closely related *S. aureus* isolates may reveal heterogeneous exoproteome profiles.^38,39^ Taken together, our combined observations imply that both qualitative and quantitative differences in the exoproteome profile might serve as markers to discriminate the 3 groups of *S. aureus* isolates with different epidemiological backgrounds. Of note, some observed differences for accessory virulence genes that are apparently distinctive for the CA and HA isolates at the genome level, such as the genes for PVL and enterotoxins, were not reflected in the present proteomics analyses, because the respective proteins were not detected. This lack of detection may relate to their actual expression levels under the investigated conditions, or to the fact that the actual identification of proteins by MS depends on various factors, including the method of sample preparation, or the acquisition and analysis of the MS data. Yet, it should be noted that these proteins were identified in some other proteomic studies.^40,41^

Knowledge of the sub-cellular localization of bacterial proteins can provide valuable insights into protein functions, especially in relation to colonization of the host, fitness and virulence.^26^ In this respect, the attention is usually focused on actively secreted proteins that are synthesized with N-terminal signal peptides, because these include the major known virulence factors.^26,27^ However, our proteomic analysis revealed only few significant differences in the detection of actively secreted proteins in the CA and HA groups that could be related to virulence (i.e., IsdA and IsdB), adhesion to host tissues (i.e., Ebps, Vwb), or immune evasion (i.e., SCIN). In addition, some other known virulence factors, such as CHIPS, the enterotoxin type D and the enterotoxin type A were uniquely identified in the CA^DK^, HA^DK^, and HA^NLDE^ isolates, respectively, irrespective of the growth phase.On the other hand, we observed major differences in the appearance of predicted cytoplasmic proteins in the exoproteomes of the investigated CA and HA isolates, where a higher number of cytoplasmic proteins was identified in the growth medium of the CA group than in the medium of the 2 HA groups during the exponential growth phase. Conversely, a higher number of cytoplasmic proteins was identified in the HA group than the CA group during the stationary growth phase. This difference can be interpreted in at least 3 ways. Firstly, there may be a difference in the timing of autolysis of cells^36,37^ resulting in the early release of cytoplasmic proteins into the extracellular milieu by cells from the CA group. On the other hand, it is known that cell wall-associated and secreted proteases can degrade cytoplasmic proteins released into the growth medium.^36^ Hence a second possible explanation for the observed differences would be that the HA isolates are more proteolytic in the exponential growth phase than the CA isolates, leading to the observed differences due to degradation of liberated cytoplasmic proteins. In a third possible scenario, ‘cytoplasmic’ proteins are actively delivered into the growth medium via non-classical secretion mechanisms that are as yet ill-defined.^33,34,37^ In this case, one would have to assume that the timing of non-classical secretion differs for CA and HA isolates. Inspection of the genome sequences of the investigated MRSA isolates showed that the genes for known autolysins (*atl, isaA, lytM*), proteases (*aur, sspA, sspB, sspC, spiA, spiB, spiC, spiD, spiE, spiF* and *IsaA*) and secretion pathways (*sec, tat* and type VII secretion) are intact with only few SNPs detectable in the coding and intergenic regions, as was the case for major gene regulators. None of the observed SNPs causes a premature stop of translation or a mutation that is known to be important for activity (data not shown). Of note, our comparative genome analysis suggests that the distinctive features of CA- and HA-MRSA isolates relate predominantly to the accessory genome, which might suggest a role of the respective genes at least in the timing of the extracellular appearance of predicted cytoplasmic proteins.

The principal component analysis (PCA) on the exoproteome databased on normalized spectral counts grouped the 15 investigated *S. aureus* isolates into 2 distinct groups. Herein, all the HA isolates formed a distinct cluster, whereas 4 of the 6 CA isolates formed a separate cluster. Importantly, the clustering of all HA^DK^ and HA^NL-DE^ isolates in one group implies that our proteomics approach identifies a common signature of all investigated HA-MRSA isolates. Intriguingly, 2 isolates designated as CA grouped with the HA isolates, suggesting that these 2 “CA isolates” (D29, D61) may actually be hospital-adapted isolates that could have been propagated in the community. Consistent with this idea, our Northern blotting analysis showed that the transcript levels for exoproteins like FabF, RpoB and PSMα1–4 in the CA isolate D29 resembled more closely the respective profiles in the HA isolates. Yet, this was not the case for isolate D61, whose *fabF* and *rpoB* transcript levels matched with those of the CA isolates, while the *psmα1–4* transcript level was very low. An alternatively possibility is that the CA isolates D29 and D61 are genuine CA isolates, in which case our proteome analyses might highlight another distinguishing feature of the 2 clusters. Indeed, the distinction of CA and HA groups based on our PCA analysis seems to have predictive value for the growth behavior and survival of *S. aureus* in non-professional phagocytic epithelial cells. Clearly, the CA isolates displayed an increase in net cell number after internalization by epithelial cells compared with the HA isolates, whose bacterial count did not substantially increase following internalization. This finding would be fully in line with an earlier study that suggested better survival of CA-MRSA than of HA-MRSA inside human neutrophils.^42^ Together, these findings imply that the cytoplasmic proteins identified in the exoproteome are indicative not only for the epidemiological behavior, but also could have an impact on the intracellular behavior of *S. aureus* within epithelial cells influencing their survival or replication capabilities. Of course, this does not exclude the possible involvement of known virulence factors with a clear role in virulence, such as phenol-soluble modulins, which were previously implicated in epidemiological behavior and intracellular survival,^29,43-45^ or the leukocidins PVL or LukAB/ED.^46^

In recent years, increasing evidence has been obtained that particular cytoplasmic proteins may have different functions at intracellular and extracellular locations.^47,48^ Such proteins are often regarded as ‘moonlighting’ proteins. Of note, the cytoplasmic proteins which we could consider here as potential moonlighting proteins are mostly proteins that are constitutively expressed at relatively high levels, and that have previously identified roles in processes such as sugar metabolism, adherence to host tissues, pathogenesis, and/or immune evasion.^49-52^ Of note, several of these potentially moonlighting proteins do not only occur in prokaryotes but also in eukaryotes, which could be suggestive of molecular mimicry where pathogens disguise themselves with a corona of factors that the human immune system does not recognize as non-self.^50,53,54^ Altogether, the possible roles of moonlighting cytoplasmic proteins in the different epidemiology and intracellular survival of CA and HA USA300 isolates as highlighted in our present study would be fully in line with recent studies where it was proposed that such proteins contribute to staphylococcal virulence.^33,35,55,56^

We conclude that our present proteomics approach to identify exoproteome signatures, and the results obtained with this approach, open up novel avenues to study and predict the epidemiological behavior of clinical MRSA isolates. Clearly, our study is built on genetically closely related MRSA isolates with distinct epidemiological behavior. It will be an important challenge for future research to assess whether a similar distinction can be achieved when this approach is applied to genetically distantly related MRSA isolates with different epidemiological behavior in hospitals and the community.

## Materials and methods

### Bacterial isolates

Relevant properties of the 15 MRSA isolates used for exoproteome analyses are listed in Table S7. 12 isolates with the PFGE profile USA300 were collected by the Statens Serum Institut (Copenhagen, Denmark) in the period between 1999 and 2006.^21^ These 12 isolates included 6 CA-MRSA isolates with *spa* type t008 (referred to as CA^DK^), and 6 HA-MRSA isolates with *spa* type t024 (referred to as HA^DK^).^18,21^ The remaining 3 HA-MRSA isolates with *spa* types t008 or t024 (referred to as HA^NL-DE^ isolates) were collected in the period between 1996 to 2010 in hospitals located within the Dutch-German border region (EUREGIO).^19^

### Whole genome sequencing of isolates and analysis

Whole genome sequencing of the investigated *S. aureus* isolates was performed on an Illumina MiSeq instrument and the Nextera® XT, 2 × 250bp kit using the manufacturer's standard protocols (Illumina, Inc., USA). DNA for the sequencing was extracted using the DNeasy Blood and Tissue kit (Qiagen, Valencia, CA, USA). The WGS data sets generated and/or analyzed in the current study are available in the European Nucleotide Archive (ENA) repository under accession number ERP018940 (http://www.ebi.ac.uk/ena/data/view/PRJEB17079). Reads from the isolates were assembled using SPAdes,^57^ annotated using PROKKA,^58^ and the core and pan-genome of the isolates was estimated using ROARY.^59^ The alignment of the core genome from ROARY was used as input to create a phylogenetic tree using RAxML^60^ with 100 bootstrap supports. The phylogenetic tree and accessory genome heatmap were visualized using ggtree. Specifically, the phylogenetic tree was based on the variable positions in the core genome of the 15 isolates (2,334 genes). The accessory genome was defined as genes present in at most 70% of the isolates resulting in 992 accessory genes. Virulence genes were identified using VirulenceFinder.^61^ The analysis of potential antimicrobial resistance genes was performed with the web-based ResFinder tool.^62^

### Bacterial cultivation for proteome sampling

Bacteria were grown overnight (14–16 h) at 37°C in 25 mL Trypton Soy Broth (TSB) under vigorous shaking (115 rpm). The cultures were then diluted into 25 mL pre-warmed RPMI 1640 medium supplemented with 2 mM glutamine (GE Healthcare/PAA, Little Chalfont, United Kingdom) to an OD_600_ of 0.05 and cultivation was continued under the same conditions. Exponentially growing cells with an OD_600_ of ∼0.5 were re-diluted into 120 mL fresh, pre-warmed RPMI medium to a final OD_600_ of 0.05. The cultivation was continued until the cultures had reached 90 min within the stationary growth phase. Within this period, 2 time points were selected for sample collection. The first one was set at OD_600_ of ∼0.5, corresponding to the exponential growth phase, and the second one was set at ∼OD_600_ of 1.3, corresponding to approx. 90 min after entry into the stationary phase. At these 2 time points, 1.5 ml culture aliquots were collected. In brief, the collected aliquots were centrifuged for 10 min at 4°C and 8000 × g with the subsequent application of a 0.22 μM filter step (GE Healthcare Systems, Little Chalfont, United Kingdom) to remove the remaining bacterial cells. The extracellular proteins in the supernatant were precipitated with 10% w/v TCA on ice at 4°C overnight. Finally, the precipitates were collected by centrifugation for 20 min at 4°C and 8000 × g, washed with ice-cold acetone, and dried at room temperature. The dried protein pellets were stored at −20°C until further use. For each isolate 2 biologic replicates were analyzed, which adds up to 24 exponential phase and 24 stationary phase samples for the CA^DK^ and HA^DK^ strains, respectively, and to 6 exponential phase and 6 stationary phase samples for the HA^NL-DE^ strains.

### Sample preparation for proteome analysis

Dried protein samples were processed as described previously.^54^ Briefly, protein pellets were dissolved in 50 mM ammonium bicarbonate buffer (Fluka, Buchs, Switzerland), reduced with 10 mM dithiothreitol (DuchefaBiochemie, Haarlem, the Netherlands) for 30 min, and alkylated with 10 mM iodoacetamide (Sigma-Aldrich, St. Louis, USA) for 30 min in the dark. To digest complex protein samples, 80 ng trypsin (Promega, Madison, USA) was added and the samples were incubated overnight at 37°C under static conditions. To stop the digestion, the samples were acidified with a final concentration of 0.1% trifluoroacetic acid (TFA, Sigma-Aldrich, St. Louis, USA) and subsequently purified using ZipTips (Millipore, Billerica, USA). For this purpose, the tips were stepwise equilibrated with 30 μL acetonitrile (ACN, Fluka, Buchs, Switzerland), 30 μL 80% ACN/0.1% TFA, 50% ACN/0.1% TFA, 30 μL 30% ACN/0.1% TFA and finally 30 μL 0.1% TFA. Peptides were bound to ZipTips by pipetting 10 times 10 μL of the sample. Impurities were removed by washing with 50 μL 0.1% TFA and finally peptides were eluted with 20 μL 50% ACN/0.1% TFA and 20 μL 80% ACN/0.1% TFA. The final eluates were concentrated using a vacuum centrifuge (Eppendorf, Hamburg, Germany) and stored at 4°C until further use.

### Mass spectrometry

Tryptic peptides were separated by reversed phase liquid chromatography (LC) coupled online to electrospray ionization mass spectrometry (ESI-MS) using an LTQ Orbitrap as described by Stobernack et al.^63^ Database searching was done with Sorcerer-SEQUEST 4 (Sage-N Research, Milpitas, USA). After extraction from the raw files, *.dta files were searched with Sequest against a target-decoy database with a set of common laboratory contaminants. The databases for the respective peptide/protein search were created from the genome sequences of the 15 investigated MRSA isolates. The RAST annotation file of these 15 MRSA isolates was used to create a non-redundant database comprising protein sequences of all isolates. Protein sequences that differed in only one amino acid were included in this database. Finally, validation of MS/MS-based peptide and protein identifications was performed with Scaffold v4.3.4 (Proteome Software, Portland, USA). Peptide identifications were accepted if they exceeded specific database search engine thresholds. SEQUEST identifications required at least deltaCn scores of greater than 0.1 and XCorr scores of greater than 2.2, 3.3 and 3.75 for doubly, triply and quadruply charged peptides, respectively. With these filter parameters, no false-positive hits were obtained, which was verified by a search against a concatenated target-pseudo reversed decoy database. Normalized spectral counts were obtained from the Scaffold file by considering a 99% protein threshold, and a minimum of 2 peptides for each protein. The normalized spectral count data were exported from Scaffold and curated in Microsoft Excel before further analysis. The filtered MS data associated with this manuscript can be downloaded from the PRIDE partner repository of the ProteomeXchange Consortium using the following link: http://www.ebi.ac.uk/pride/archive/login.

### Prediction of protein localization, biologic processes and molecular functions

Prediction of the subcellular localization of proteins that were identified by LC-MS/MS was performed using different bioinformatics tools. Since individual bioinformatics tools are not able to specifically predict all possible localization sites of bacterial proteins,^64^ we used 8 different computer programs, namely SignalP4.1 (http://www.cbs.dtu.dk/Services/SignalP/),^65^ Phobius (http://www.ebi.ac.uk/Tools/pfa/phobius/),^66^ Predisi (http://www.predisi.de/),^67^ LipoP1.0 (http://www.cbs.dtu.dk/services/LipoP),^68^ ProtCompB 9.0 (http://linux1.softberry.com/berry.phtml?topic=pcompb&group=programs&subgroup=proloc),^69^ PSORTb v 3.0.3 (http://www.psort.org/psortb/index.html),^70^ TMHMM2.0c (http://www.cbs.dtu.dk/services/TMHMM),^71^ and CDD-batch search (https://www.ncbi.nlm.nih.gov/Structure/bwrpsb/bwrpsb.cgi).^72^ The settings used for of each program are specified in Table S8. A detailed description of output parameters, scores and thresholds for each tag is presented in Table S9. Voronoi treemaps to link quantitative proteomic data and functional classifications were created using the Paver software (DECODON GmbH, Greifswald, Germany) with the latest functional categorization of SEED database of *S. aureus* USA300_FPR3757.^73^

### RNA isolation

Bacterial isolates were grown under the same condition as for the proteomics sample collection. 25 ml culture aliquots were collected for RNA isolation 90 min after entry into the stationary growth phase, corresponding to an OD_600_ of approx. 1.3. RNA was isolated from bacteria as described previously.^74^ Briefly, ½ volume of frozen killing buffer (20 mM Tris/HCl [pH 7.5], 5 mM MgCl2, 20mM NaN3) was added to the bacterial culture, and bacterial cells were collected by centrifugation for 3 minutes, 8000 rpm at 4°C. The supernatant was discarded, and pellets were frozen in liquid nitrogen and stored at −80°C until further processing. Cell pellets were re-suspended in ice-cold killing buffer and transferred into Teflon vessels filled with liquid N_2_ for disruption. Cells were then mechanically disrupted with a Mikro-Dismembrator S (Sartorius) for 2 min, 2600 rpm. The resulting powder was re-suspended in lysis solution that was pre-warmed at 50°C (4 M guanidine thiocyanate, 25 mM sodium acetate [pH 5.2], 0.5% N-laurylsarcosinate 40 [wt/vol]) by repeated up- and down-pipetting. Then, lysates were transferred into pre-cooled micro-centrifuge tubes, and frozen at −80°C.

Total RNA was isolated by phenol-chloroform extraction as described previously.^74^ Samples were processed twice with an equal volume of acid phenol solution (Sigma-Aldrich, Zwijndrecht, the Netherlands), and mixed thoroughly on an Eppendorf tube shaker until completely thawed. The resulting suspension was then centrifuged for 5 min, 12000 rpm, and the supernatant was transferred into a fresh microcentrifuge tube. Next, samples were processed once with one volume of Chloroform/isoamyl alcohol, mixed well, and centrifuged for 5 min, 12000 rpm. RNA was precipitated from the supernatant by the addition of 1/10 volume of 3 M Na-Acetate, pH 5.2, and 0.8 ml of isopropanol. The precipitated RNA was washed once with 70% RNase-free ethanol, and dissolved in RNase-free water.

### Northern blot analysis

Northern blot analysis was performed as described previously.^75^ Specific biotin-labeled RNA probes were generated by *in vitro* synthesis using a T7 RNA polymerase and Bio-16-UTP (Life Technologies). 3–10 μg of total RNA per lane was separated on 1.2% denaturing agarose gels. Gene-specific transcripts were detected with the aid of biotin-labeled anti-sense RNA-probes. Fluorescent detection of the biotin- labeled probes was performed using IRDye® 800CW Streptavidin (LI-COR Biosciences - GmbH) and the Odyssey Clx Imaging System (LI-COR Biosciences - GmbH) according to the instructions of the manufacturer. Primer sequences are listed in Table S10.

### Survival of bacteria upon epithelial cell infection

#### Cell lines and culture conditions

The human bronchial epithelial cell line 16HBE14o- was used to investigate the survival of MRSA isolates upon internalization. The epithelial cells were cultured in eukaryotic minimal essential medium (eMEM; 1x MEM without arginine and lysine; Costumer formulation, PromoCell GmbH, Heidelberg, Germany) supplemented with 10% (v/v) fetal calf serum (FCS; Biochrom AG, Berlin, Germany), 2% (v/v) L-glutamine 200 mM (PAN-Biotech GmbH, Aidenbach, Germany) and 1% (v/v) non-essential amino acids 100x (PAN-Biotech GmbH). The cells were seeded at a density of 1 × 10^5^ cells/cm^2^ in CellStar® 12-well plates (Greiner Bio-One, Frickenhausen, Germany) and cultured for 3 d at 37°C, 5% CO_2_ in a humidified atmosphere after which they were ready for infection experiments.

#### Bacterial culture conditions

The bacteria were cultured in prokaryotic minimal essential medium (pMEM; 1x MEM without sodium bicarbonate; Invitrogen, Karlsruhe, Germany) supplemented with 1x non-essential amino acids (PAN-Biotech GmbH), 4 mM L-glutamine (PAN-Biotech GmbH), 10 mM HEPES (PAN-Biotech GmbH), 2 mM L-alanine, 2 mM L-leucine, 2 mM L-isoleucine, 2 mM L-valine, 2 mM L-aspartate, 2 mM L-glutamate, 2 mM L-serine, 2 mM L-threonine, 2 mM L-cysteine, 2 mM L-proline, 2 mM L-histidine, 2 mM L-phenyl alanine and 2 mM L-tryptophan (Sigma-Aldrich, Munich, Germany), adjusted to pH 7.4 and sterilized through filtration. Notably, for the overnight pre-culture 0.01% of yeast extract was added.

#### Internalization procedure

The internalization of MRSA into epithelial cells was performed as described previously by Pförtner et al.^76^ Briefly, bacterial cultures were inoculated from exponentially growing overnight cultures, starting at an inoculation OD_600_ of 0.05 and permitting growth until the mid-exponential phase at 37°C, 150 rpm in a shaking water bath. The bacterial numbers were determined by flow cytometry with a Guava easyCyte™ flow cytometer (MilliporePrior Billerica, MA, USA). Prior to infection, the numbers of epithelial cells were assessed by detaching them from the plates with trypsin-EDTA 0.25% (Thermo Fisher Scientific, Waltham, USA), mixing with Trypan blue dye, and counting with a Countess® cell counter (Invitrogen, Karlsruhe, Germany). To infect epithelial cells with MRSA at a multiplicity of infection (MOI) of 1:25, the host cell medium was exchanged with the infection mix (MRSA diluted on eMEM, buffered with 2.9 µl sodium hydrogen carbonate [7.5%] per ml of bacterial culture added) and incubated for one hour at 37°C, 5% CO_2_ in an incubator. Afterwards, the cell culture medium was exchanged with fresh eMEM containing 10 µg/ml lysostaphin, and this medium was exchanged every 2 d for long-term experiments.

Sampling of the 16HBE14o- cells was performed by detaching of the cells from the plate with trypsin-EDTA 0.25%, and the collection of internalized bacteria was performed through incubation of the plate with 0.05% SDS for 5 min. Quantification of the intracellular MRSA isolates was performed by flow cytometry with a GUAVA®easyCyte (Merck Millipore, Darmstadt, Germany). To this end, the bacteria were stained with 0.2 µg/ml Vancomycin BODIPY FL (Thermo Fisher Scientific, Waltham, USA), and detected using a 488 nm laser for excitation as described.^77^ The intracellular survival of each isolate was analyzed in independent duplicate experiments.

### Graphical and statistical analyses

Volcano plot analyses were performed using GraphPad Prism version 6. Statistical analyses were performed using the Wilcoxon signed-rank test. A P-value of less than or equal to 0.05 was considered statistically significant. Principal component analysis (PCA) was performed using the Statistical Package for Social Science (SPSS) version 22. The component loading of the extracellular proteins from the 15 CA^DK^, HA^DK^ and HA^NL-DE^ isolates was calculated both for growth medium fractions of cells in the exponential and stationary growth phases based on normalized spectral count. The Venn diagram was constructed using Venny version (http://bioinfogp.cnb.csic.es/tools/venny/).

## Supplementary Material

KVIR_S_1325064.zip
