# Supplementary material for: Signatures of cytoplasmic proteins in the exoproteome distinguish community- and hospital-associated methicillin-resistant Staphylococcus aureus USA300 lineages
Source: Virulence. 2017 May 5;8(6):891–907. doi: 10.1080/21505594.2017.1325064 (PMC5626246; doi:10.1080/21505594.2017.1325064)
Supplement: KVIR_S_1325064.zip [file kvir-08-06-1325064-s001.zip › KVIR_S_1325064_Fig 1.pdf]

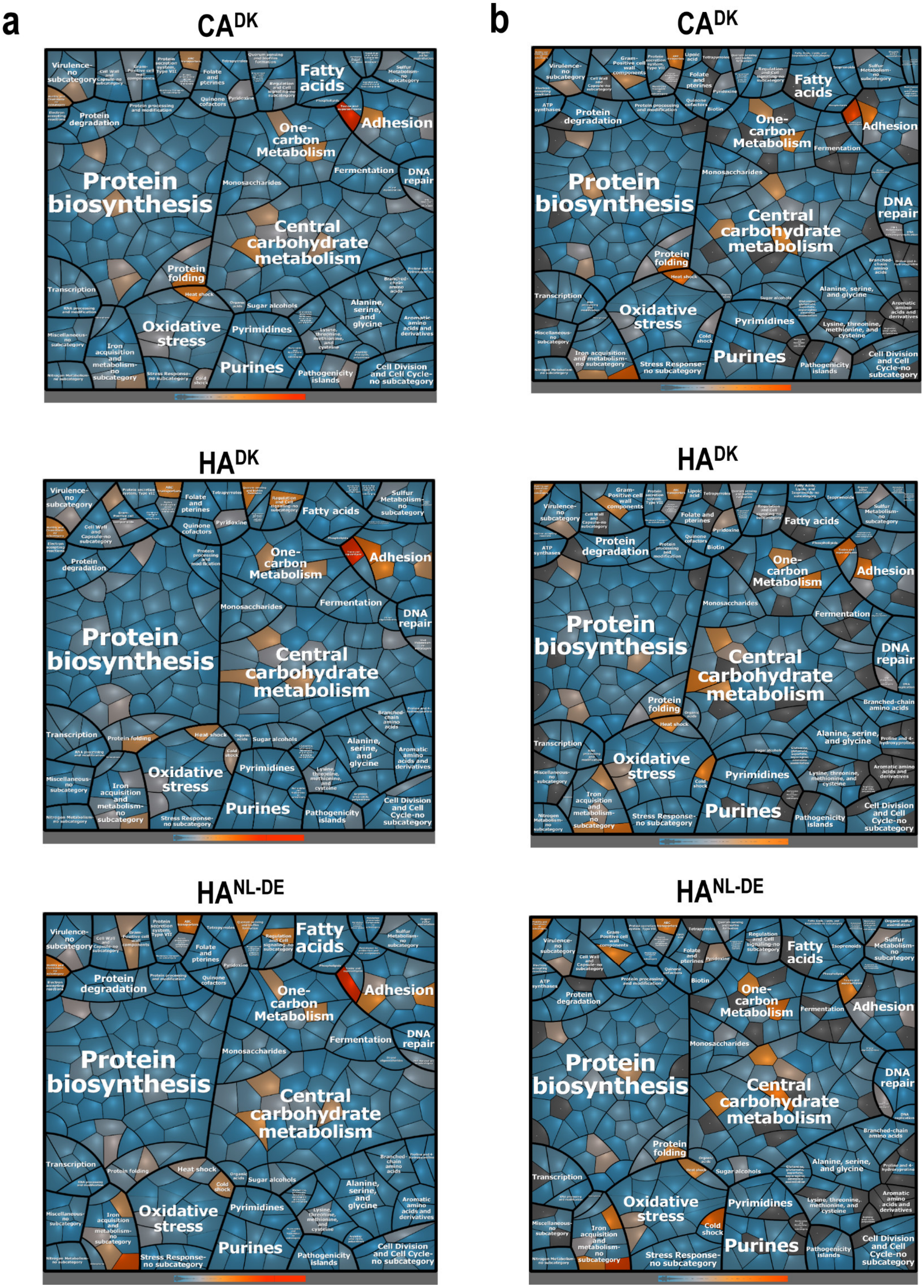
a
b
CA^{DK}
HA^{DK}
HA^{NL-DE}
Protein biosynthesis
Central carbohydrate metabolism
One-carbon Metabolism
Adhesion
Fatty acids
Protein degradation
Protein folding
Heat shock
Oxidative stress
Purines
Pyrimidines
DNA repair
Fermentation
Monosaccharides
Transcription
Sugar alcohols
Alanine, serine, and glycine
Aromatic amino acids and derivatives
Branched-chain amino acids
Lysine, threonine, methionine, and cysteine
Pathogenicity islands
Cell Division and Cell Cycle-no subcategory
Stress Response-no subcategory
Iron acquisition and metabolism-no subcategory
Miscellaneous-no subcategory
Virulence-no subcategory
Folate and pterines
Quinone cofactors
Pyridoxine
Sulfur Metabolism-no subcategory
Cold shock
Biotin
ATP synthases
Isoprenoids
Protein processing and modification
Cell Wall and Capsule-no subcategory
Gram-Positive cell wall components
Regulation and Cell signaling-no subcategory
Nitrogen Metabolism-no subcategory

Supplementary Figure 1. Voronoi treemap of functional categories for the identified proteins. Spectral count values representing the abundance levels of proteins from the investigated CA^{DK}, HA^{DK} and $HA^{NL\text{-}DE}$ isolates are color-coded (blue for low abundance; red for high abundance). Proteins not identified in the particular isolate group or condition are labeled in black. (**a**) Proteins in growth medium fractions from cultures in the exponential growth phase, or (**b**) from cultures in the stationary growth phase.
