# Supplementary material for: Signatures of cytoplasmic proteins in the exoproteome distinguish community- and hospital-associated methicillin-resistant Staphylococcus aureus USA300 lineages
Source: Virulence. 2017 May 5;8(6):891–907. doi: 10.1080/21505594.2017.1325064 (PMC5626246; doi:10.1080/21505594.2017.1325064)
Supplement: KVIR_S_1325064.zip [file kvir-08-06-1325064-s001.zip › KVIR_S_1325064_Fig 2.pdf]

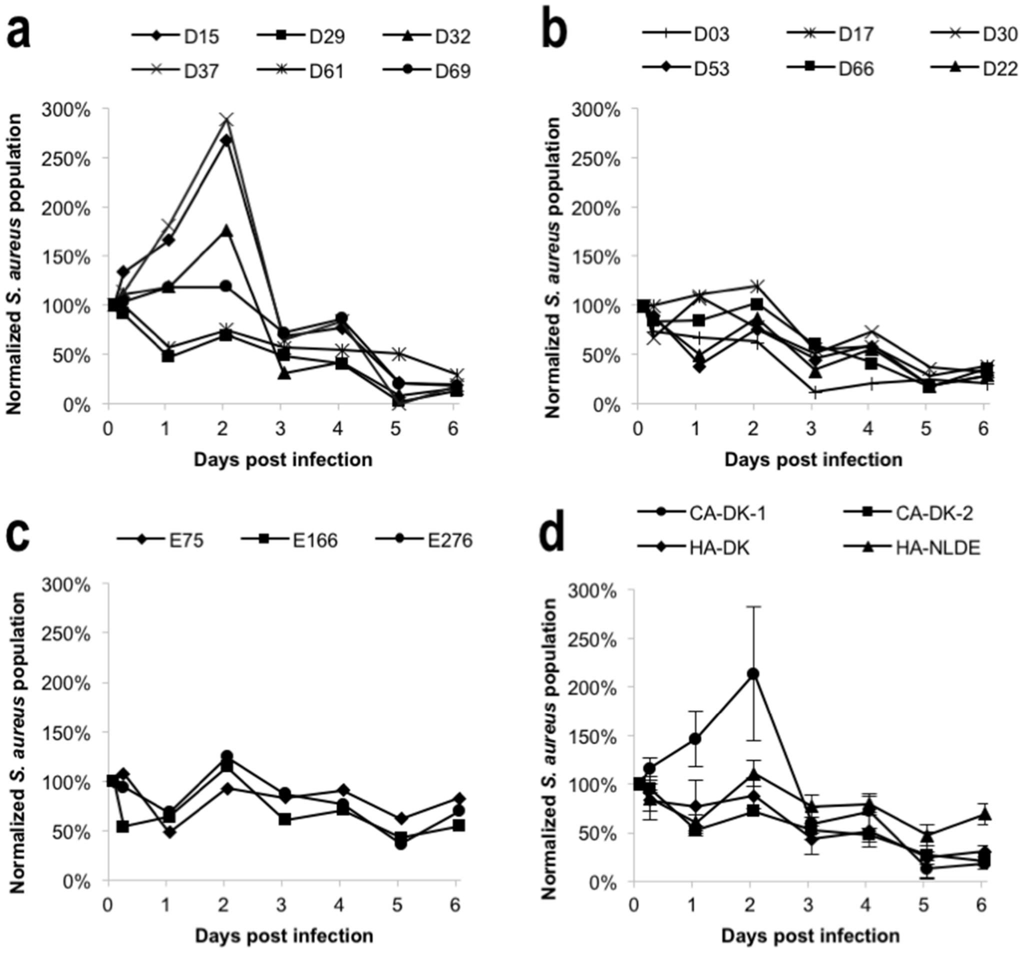

Supplementary Figure 2. Survival of CA and HA isolates internalized by epithelial cells

The 16HBE14o- bronchial epithelial cell line was used to investigate the intracellular survival of CA and HA isolates. A multiplicity of infection (MOI) of 1:25 was used and bacterial survival was assayed over a period of six days. The quantification of intracellular MRSA survival was performed by staining bacterial samples with 0.2 μg/ml vancomycin BODIPY FL and counting by flow cytometry. The intracellular survival of each isolate was analyzed in independent duplicate experiments. Survival curves are shown for the (**a**) CA^{DK} isolates, (**b**) HA^{DK} isolates, and (**c**) $HA^{NL\text{-}DE}$ isolates. (**d**) Averaged survival curves for the CA^{DK}, HA^{DK} and $HA^{NL\text{-}DE}$ isolates, where the CA^{DK} isolates are separated into two groups in accordance with their exoprotein abundance signatures in **Fig. 8** (CA-DK-1 includes isolates D15, D32, D37 and D69; CA-DK-2 includes isolates D29 and D61).
