# Supplementary material for: Signatures of cytoplasmic proteins in the exoproteome distinguish community- and hospital-associated methicillin-resistant Staphylococcus aureus USA300 lineages
Source: Virulence. 2017 May 5;8(6):891–907. doi: 10.1080/21505594.2017.1325064 (PMC5626246; doi:10.1080/21505594.2017.1325064)
Supplement: KVIR_S_1325064.zip [file kvir-08-06-1325064-s001.zip › KVIR_S_1325064_Table 6.docx]

**Supplementary Table 6: Principal component analysis (PCA) of the normalized spectral counts of identified extracellular proteins.** Computation of the component loading for the different extracellular proteins based on their predicted subcellular localization.

| **Isolates** | **Components^a^** | |  | **Component^b^** |  | **Component^c^** |  | **Components^d^** | |
| --- | --- | --- | --- | --- | --- | --- | --- | --- | --- |
|  | 1 | 2 |  |  |  |  |  | 1 | 2 |
| D3 | 0.806 | 0.52 |  | 0.945 |  | 0.076 |  | 0.797 | 0.54 |
| D17 | 0.761 | 0.56 |  | 0.94 |  | 0.076 |  | 0.632 | 0.696 |
| D22 | 0.824 | 0.424 |  | 0.948 |  | 0.076 |  | 0.484 | 0.764 |
| D30 | 0.814 | 0.429 |  | 0.881 |  | 0.071 |  | 0.805 | 0.488 |
| D53 | 0.904 | 0.371 |  | 0.952 |  | 0.077 |  | 0.856 | 0.455 |
| D66 | 0.749 | 0.586 |  | 0.946 |  | 0.076 |  | 0.775 | 0.565 |
| D15 | 0.411 | 0.865 |  | 0.898 |  | 0.072 |  | 0.317 | 0.875 |
| D29 | 0.739 | 0.391 |  | 0.751 |  | 0.06 |  | 0.839 | 0.48 |
| D32 | 0.478 | 0.823 |  | 0.907 |  | 0.073 |  | 0.331 | 0.905 |
| D37 | 0.399 | 0.893 |  | 0.929 |  | 0.075 |  | 0.363 | 0.897 |
| D61 | 0.606 | 0. 625 |  | 0.964 |  | 0.078 |  | 0.895 | 0.261 |
| D69 | 0.354 | 0.896 |  | 0.917 |  | 0.074 |  | 0.431 | 0.819 |
| E75 | 0.759 | 0.539 |  | 0.923 |  | 0.074 |  | 0.89 | 0.405 |
| E166 | 0.832 | 0.347 |  | 0.832 |  | 0.067 |  | 0.921 | 0.324 |
| E276 | 0.842 | 0.309 |  | 0.888 |  | 0.072 |  | 0.571 | 0.669 |

Component**^a^**, all proteins; component**^b^**, cytoplasmic proteins removed; component**^c^**, cytoplasmic and unknown proteins removed; component**^d^**, only cytoplasmic proteins.
