# Supplementary material for: Signatures of cytoplasmic proteins in the exoproteome distinguish community- and hospital-associated methicillin-resistant Staphylococcus aureus USA300 lineages
Source: Virulence. 2017 May 5;8(6):891–907. doi: 10.1080/21505594.2017.1325064 (PMC5626246; doi:10.1080/21505594.2017.1325064)
Supplement: KVIR_S_1325064.zip [file kvir-08-06-1325064-s001.zip › KVIR_S_1325064_Table 7.docx]

**Supplementary Table 7: *S. aureus* isolates used in this study.**

| **Name of isolate** | **Year of isolation** | **Age of carrier** | **Area of residence** | **Epidemio-logical behavior** | ***spa*-type** | **SCC*mec*** | **PVL** | **ACME** |
| --- | --- | --- | --- | --- | --- | --- | --- | --- |
| D15 | 2004 | 39 | Denmark | CA | t008 | IV | + | + |
| D29 | 2004 | 40 | Denmark | CA | t008 | IV | + | + |
| D32 | 2005 | 61 | Denmark | CA | t008 | IV | + | + |
| D37 | 2005 | 24 | Denmark | CA | t008 | IV | + | + |
| D61 | 2005 | 5 | Denmark | CA | t008 | IV | + | + |
| D69 | 2005 | 26 | Denmark | CA | t008 | IV | + | + |
| D3 | 2003 | 48 | Denmark | HA | t024 | IV | - | - |
| D17 | 2004 | 82 | Denmark | HA | t024 | IV | - | + |
| D22 | 2004 | 79 | Denmark | HA | t024 | IV | - | + |
| D30 | 2004 | 88 | Denmark | HA | t024 | IV | - | + |
| D53 | 2005 | 65 | Denmark | HA | t024 | IVa | - | + |
| D66 | 2005 | 61 | Denmark | HA | t024 | IVa | - | + |
| E75 | 2005 | unknown | Dutch-German region | HA | t008 | IV | - | - |
| E166 | 2006 | unknown | Dutch-German region | HA | t008 | IV | - | - |
| E276 | 2007 | unknown | Dutch-German region | HA | t024 | IV | - | - |

*CA= community associated, HA= hospital associated
