# Supplementary material for: Signatures of cytoplasmic proteins in the exoproteome distinguish community- and hospital-associated methicillin-resistant Staphylococcus aureus USA300 lineages
Source: Virulence. 2017 May 5;8(6):891–907. doi: 10.1080/21505594.2017.1325064 (PMC5626246; doi:10.1080/21505594.2017.1325064)
Supplement: KVIR_S_1325064.zip [file kvir-08-06-1325064-s001.zip › KVIR_S_1325064_Table 8.docx]

**Supplementary Table 8:** **Setting of programs used for predicting the subcellular localization of identified extracellular proteins.**

| Program | Parameters | Value | |
| --- | --- | --- | --- |
| SignalP 4.1 | Organism group | Gram+ | |
|  | D-cutoff (noTM network) | 0.57 | |
|  | D-cutoff (TM network) | 0.45 | |
|  | Truncated at a.a. # | 100 | |
|  | Method | Input sequences may include TM regions | |
|  | Min. predicted SP length | 10 a.a. | |
| Phobius | No possible parameters | | |
| LipoP 1.0a | No possible parameters | | |
| TMHMM 2.0c | No possible parameters | | |
| PrediSi | Organism group | Gram+ | |
| PSortb 3.0.3 | Organism group | Gram+ | |
|  | Cutoff | 7.5 (default) | |
|  | Divergent | 7.5 (default) | |
| ProtCompB | Organism group | Gram+ | |
| CDD-batch search | List of superfamilies to look for | cl06526  cl00046  cl02763  cl22949  cl07020  cl06736  cl03229  cl04409  cl19379  cl07731  cl21614  cl21621  cl17055  cl06507  cl21525  cl06814  cl18368  cl23125  cl17036  cl15985  cl02857  cl20286  cl22626 | cl23671  cl23502  cl02760  cl02712  cl06494  cl04270  cl21534  cl09098  cl16279  cl20286  cl02713  cl15580  cl01439  cl11079  cl03820  cl11852  cl14016  cl03487  cl16280  cl15692  cl07893  cl15753  cl05349  cl01709 |
