# Supplementary material for: Signatures of cytoplasmic proteins in the exoproteome distinguish community- and hospital-associated methicillin-resistant Staphylococcus aureus USA300 lineages
Source: Virulence. 2017 May 5;8(6):891–907. doi: 10.1080/21505594.2017.1325064 (PMC5626246; doi:10.1080/21505594.2017.1325064)
Supplement: KVIR_S_1325064.zip [file kvir-08-06-1325064-s001.zip › KVIR_S_1325064_Table 9.docx]

**Supplementary Table 9:** **Conditions applied for score and tag attribution with the programs applied to predict the subcellular localization of identified extracellular proteins.**

| Tag | Program | Output parameter | Condition | Score |
| --- | --- | --- | --- | --- |
| SEC | PrediSi | SP | Yes | +1 |
| Threshold > 5 | SignalP | SP | Yes | +1 |
| (out of 8) | Phobius | #TM | < 3 | +1 |
|  |  | SP | Yes | +1 |
|  | LipoP | Prediction | SpI | +1 |
|  | TMHMM | #TM | < 3 | +1 |
|  | Psortb | Prediction | Secreted (XOR) or | +1 |
|  |  |  | Cellwall (XOR) | +1 |
|  | ProtCompB | Prediction | Secreted | +1 |
| CYT | PrediSi | SP | No | +1 |
| Threshold > 5 | SignalP | SP | No | +1 |
| (out of 8) | Phobius | #TM | < 1 | +1 |
|  |  | SP | No | +1 |
|  | LipoP | Prediction | Cyt | +1 |
|  | TMHMM | #TM | < 1 | +1 |
|  | Psortb | Prediction | Cytoplasmic | +1 |
|  | ProtCompB | Prediction | Cytoplasmic | +1 |
| TM | Phobius | #TM | 0 < TM < 3 (XOR) | +1 |
| Threshold > 3 |  |  | > 2 (XOR) | +2 |
| (out of 7) | LipoP | Prediction | TMH | +1 |
|  | TMHMM | #TM | 0 < TM < 3 (XOR) | +1 |
|  |  |  | > 2 (XOR) | +2 |
|  | Psortb | Prediction | CytoplasmicMembrane | +1 |
|  | ProtCompB | Prediction | Membrane | +1 |
| LIPO | LipoP | Prediction | SpII | +1 |
| Threshold > 0 |  |  |  |  |
| (out of 1) |  |  |  |  |
| CW  Threshold > 0 (out of 1) | CDD-batch search | Superfamily matches the list | > 0 matches | +1 |
